# Discounting Behavior in Problem Gambling

**DOI:** 10.1007/s10899-021-10054-x

**Published:** 2021-07-16

**Authors:** Patrick Ring, Catharina C. Probst, Levent Neyse, Stephan Wolff, Christian Kaernbach, Thilo van Eimeren, Ulrich Schmidt

**Affiliations:** 1 Kiel Institute for the World Economy, Kiel, Germany; 2 Department of Neurology, Kiel University, Kiel, Germany; 3 SOEP, DIW, Berlin, Germany; 4 WZB, Berlin, Germany; 5 IZA, Bonn, Germany; 6 Department of Radiology and Neuroradiology, Kiel University, Kiel, Germany; 7 Department of Psychology, Kiel University, Kiel, Germany; 8 Department of Nuclear Medicine, University Hospital Cologne, Cologne, Germany; 9 Department of Neurology, University Hospital Cologne, Cologne, Germany; 10 Department of Economics, Kiel University, Kiel, Germany; 11 Department of Economics and Econometrics, University of Johannesburg, Johannesburg, South Africa

**Keywords:** Time preferences, Discounting, Risk, Incentives, Gambling

## Abstract

Problem gamblers discount delayed rewards more rapidly than do non-gambling controls. Understanding this impulsivity is important for developing treatment options. In this article, we seek to make two contributions: First, we ask which of the currently debated economic models of intertemporal choice (exponential versus hyperbolic versus quasi-hyperbolic) provides the best description of gamblers’ discounting behavior. Second, we ask how problem gamblers differ from habitual gamblers and non-gambling controls within the most favored parametrization. Our analysis reveals that the quasi-hyperbolic discounting model is strongly favored over the other two parametrizations. Within the quasi-hyperbolic discounting model, problem gamblers have both a significantly stronger present bias and a smaller long-run discount factor, which suggests that gamblers’ impulsivity has two distinct sources.

## Introduction

Problem gambling can have severe negative consequences. For example, it can cause financial problems due to debt overload (Moghaddam et al. 2015) or mental health problems due to depression (Clarke 2006) and social isolation (Trevorrow and Moore 1998). Thus, problem gambling can impose high costs on the individual, her/his social environment and on society in general. Given the high prevalence of problem gambling, which can range between 0.5 and 7.6% worldwide (Williams et al. 2012), and its high individual and social costs, problem gambling is considered to be a critical public health concern (Lancet 2017; Wardle et al. 2019).

Behavioral economics offers frameworks and tools to study gambling-related behavior and to derive preventive policies (Newall et al. 2020a, b; Galizzi and Wiesen 2017; Galizzi 2012; Folkvord et al. 2019; Ring et al. 2018; Tomasuolo 2020). In this article, we apply concepts of behavioral economics to study temporal decision making related to problem gambling. Problem gamblers have a stronger preference for smaller, immediate rewards over larger, delayed rewards than do non-gambling controls (Petry and Casarella 1999; Petry 2001; Dixon et al. 2003; MacKillop et al. 2006, 2011). This behavior fails to maximize long-term gains and has been described as an expression of impulsivity – an important factor associated with problem gambling (Green and Myerson 2010; Petry and Madden 2010; Steel and Blaszczynski 1998; Grecucci et al. 2014). The present article aims to contribute to a better understanding of this impulsivity, which seems to be important when developing new treatment options.

Different economic models exist that can account for this tendency towards impulsivity in problem gambling. These models have different features for modeling behavioral regularities with respect to discounting and differ, for example, in their functional forms and/or their degrees of freedom (for an overview, please see Kable (2014). In this paper, our first aim is to provide a systematic comparison of various discounting models in explaining problem gamblers’ time preferences (exponential versus hyperbolic versus quasi-hyperbolic). We focus on these three models because they are the influential models for intertemporal choice in (behavioral) economics (Kable 2014). Then, we study how gamblers differ from non-gambling controls within the most favored parametrization.

While it is an established finding that gamblers are more impulsive in temporal decision making, our aim is to make several contributions to the existing literature. First, we apply the quasi-hyperbolic model to explain gamblers’ time preferences. This appears to be relevant because this model has the distinct feature of modeling impulsivity by separating it into short-term and long-term oriented components. The short-term oriented component is called the present bias, and the long-term oriented component is called the discount factor. Whether impulsivity among gamblers is driven by only one of the two components or both appears to be relevant for developing behavioral interventions. Problem gamblers might have similar long-term discount factors than non-gambling controls, but the former’s preference for realizing rewards sooner is driven by a stronger present bias. This would suggest that problem gamblers suffer from a lack of self-control. In this case, behavioral strategies that protect long-term from short-term interests could be helpful, for example commitments or personal rules (Bénabou and Tirole 2004). Alternatively, problem gamblers might have a smaller long-term discount factor without exhibiting any differences in the present bias. From a behavioral economics perspective, this would reflect a difference in preferences and not an inconsistency in behavior, such as the present bias. Here, it might be possible to emphasize the negative long-term consequence of this behavior as suggested in cognitive bias modification procedures (MacLeod and Mathews 2012). Finally, both processes might contribute to the general finding that gamblers are more impulsive.

In addition to considering a different discounting model, our experimental task is incentivized, i.e., each decision has a certain probability of being actually paid out. To the best of our knowledge, the existing literature on problem gamblers’ time preferences has applied only hypothetical payments (e.g., Petry and Casarella 1999; MacKillop et al. 2006; Dixon et al. 2003; Holt et al. 2003). There is debate over the extent to which behavior differs under real versus hypothetical payments, and no consensus or underlying theory has been reached (Kühberger et al. 2002; Grether and Plott 1979; Camerer and Mobbs 2017). With respect to gambling addiction, however, money naturally appears as an important motivation (Schüll 2012; Anselme and Robinson 2013). Together with studies revealing differences in brain activation during the anticipation and realization of real monetary outcomes in problem gamblers (Balodis et al. 2012; Linnet et al. 2010; Luijten et al. 2017), it appears unclear how their behavior changes due to real incentives in comparison to non-gambling controls. To increase the external validity of our study, we apply an incentive-compatible task in which participants’ choices have actual consequences.

Furthermore, it is important to control for individual risk preferences when eliciting discount rates, because discount rates are defined over utility flows and not flows of money (Andersen et al. 2014, 2008). In our previous work (Ring et al. 2018), we demonstrate that gamblers are more risk-seeking, and this needs to be taken into account when eliciting discount rates. We use the data on risk preferences reported in our previous study, estimate a common power utility function of the form $U(x)=x^{\alpha }$ for each individual and thereby control for the curvature of the utility function.

Our study can be summarized as follows: We present a discounting task to a sample of 25 problem gamblers, 23 habitual gamblers and 26 matched non-gambling controls. In the first step of the analysis, we compare the explanatory power of various discounting models to identify the one that provides the best fit to the data (exponential versus hyperbolic versus quasi-hyperbolic). Our findings suggest that the two-parameter quasi-hyperbolic model is strongly preferred for explaining the observed data, particularly for problem gamblers. In the second step of the analysis, we fit this model to the individual data. We observe that problem gamblers have a significantly stronger present bias and a smaller long-run discount factor, which indicates that they are more impulsive in the short and long run. Both parameters correlate significantly with the participants’ South Oaks Gambling Screen (SOGS) scores (Lesieur and Blume 1987), which is a continuous variable that captures their gambling behavior.

## Methods

### Participants

For the current study, we recruited 74 participants (mean age = 38.9 years, *SD* = 14.7). The participants were recruited via advertisements in local newspapers. We made one type of call for participants that explicitly targeted regular gamblers and one that did not target them. The calls were placed bi-weekly without any overlap. During the initial phone contact, we informed the potential participants about the general experimental procedure and excluded potential participants based on the following criteria:

- Problematic (illegal) drug consumption, i.e., drug consumption at least once a week
- A medically diagnosed history of psychiatric or neurological disorders
- Standard MRI exclusion criteria

Next, the participants were invited to the University Hospital in Kiel (Germany) for a semi-structured interview (Grant et al. 2004) to evaluate their gambling behavior. The interviews were conducted by certified psychologists and took approximately 30 min. In our sample, 25 participants fulfilled at least three of the DSM-IV-TR criteria (American Psychiatric Association 2000) for pathological gambling and can therefore be classified as problem gamblers (PG group, 4 women) (Fong et al. 2011; Weintraub et al. 2009). Furthermore, 23 participants were classified as habitual gamblers (HG group, 3 women). These participants fulfilled fewer than three of the DSM-IV-TR criteria but were gambling at least once per week. Our rationale for including this group in our study was that they are also experienced with gambling, but they do not meet the diagnostic criteria for problem gambling. In this sense, this group is a relevant control group that allows us to study a broader range of the problem gambling continuum. Additionally, 26 participants, who gambled less than once per month, were recruited as a non-gambling control group (C group, 5 women). This group of participants reported no regular gambling behavior in the present or in the past and therefore did not meet any of the DSM-IV-TR diagnostic criteria for problem gambling. Please note that DSM-IV-TR was the current diagnostic criteria when we began to prepare the study materials and the pilots. In the updated version of these diagnostic criteria (DSM-5, American Psychiatric Association 2013), the criterion for gambling-related crimes was removed because it contributed little to diagnostic accuracy (Weinstock et al. 2013; Zimmerman et al. 2006). Consequently, gambling group classifications based on DSM-IV-TR compared to DSM-5 strongly correlate (Stinchfield et al. 2016; Jiménez-Murcia et al. 2019).

All participants gave written informed consent and could decide to discontinue participation at any time. The research protocol was approved by the local ethics committee of the University Hospital in Kiel, and the study was conducted in accordance with the guidelines of the Declaration of Helsinki.

In addition to the DSM-IV-TR criteria, the participants answered the SOGS (Lesieur and Blume 1987) to obtain a continuous variable for their gambling behavior. Higher values indicate a higher probability of a gambling addiction. As expected, the PG group has the highest mean SOGS score of 8.36 ($SD=3.82$), followed by the HG group with a mean score of 3.96 ($SD=2.96$) and the C group with a mean score of 0.42 ($SD=0.99$). Because the distribution of the SOGS scores in our sample violates the normality assumption (Shapiro-Wilk test, $W = 0.86, \; p < 0.001$), and we have fewer than 30 observations per group (Moffatt 2015), a (non-parametric) Kruskal-Wallis test was used to test for significant differences in the SOGS scores among the three groups. The test indicates that the groups were significantly different with respect to their SOGS scores ($H(2)=48.41, ~p <0.001$; see Table 1). Post hoc tests after Dunn with Bonferroni correction revealed that all three groups were significantly different ($p <0.001$). All three groups were matched based on characteristics that potentially affect task performance independent of gambling behavior and that may be correlated with time/risk preferences (Harrison et al. 2002; Scharff and Viscusi 2011) such as demographic variables (age, income and education), and alcohol and cigarette consumption (Kruskal-Wallis tests, $p>0.250$; see Table 1).

**Table 1:** Means of the demographic variables, alcohol and cigarette consumption, and SOGS scores by group

	C group	HG group	PG group	*p*-value
	N=26	N=23	N=25	
Age	40.46±15.22	37.57±14.16	38.48±15.13	>0.250
Income	1778.08±1533.34	1603.17±1195.96	1323.00±813.52	>0.250
Alcohol	4.47±5.34	3.72±3.73	5.41±9.64	>0.250
Smoking	37.88±70.75	32.05±41.36	46.28±47.19	>0.250
Education	12.96±2.24	12.52±2.39	12.28±1.95	>0.250
SOGS	0.42±0.99	3.96±2.96	8.36±3.82	<0.001

Age in years; Income per month in euros; Alcohol in units (0.33 l beer, 0.2 l wine or 0.02 l liquor) per week; Smoking in cigarettes per week; SOGS, South Oaks Gambling Screen

As all variables violate the normality assumption (Shapiro-Wilk test, $p<0.001$), non-parametric tests (Kruskal-Wallis tests) were performed

After the psychological interview, the participants took part in tasks to elicit their time and risk preferences, which are described in the next subsection. The experimental session also included EEG and fMRI experiments, which are not reported here.

### Experimental Task: Discounting

First, the participants were informed that one of their choices, from either the risk or discounting task, would be randomly selected to determine their payment. If the discounting task was selected, then the payment was realized as a bank transfer to avoid strategic behavior due to different payment methods.

In the discounting task, the participants had to decide between a payment of 10 euros at an earlier data and a larger varying payment at a later date (Task 1: Today versus tomorrow, Task 2: Today versus in one month; Task 3: In one month versus in two months). An example is shown in Table 2. The participants were instructed to have a maximum of one switching point per decision task.

**Table 2:** Discounting task

Option	Option A: Today	Option B: In one month	Preferred alternative	
1	10	12	A	B
2	10	14	A	B
3	10	16	A	B
4	10	18	A	B
5	10	20	A	B

### Experimental Task: Risk Taking

In the experimental task developed by Vieider et al. (2015), the participants make repeated decisions between binary monetary lotteries and different certain monetary outcomes. The task elicits risk preferences for gain-only, loss-only and mixed lotteries. For the current article, we are interested only in the decisions made in the gain domain, and we therefore restrict the description accordingly^1^: In the pure gain domain, participants face several choice situations that involve a fixed lottery and varying certain payments. The lottery consists of two outcomes that are denoted by *x* and *y*. *x* is realized with probability *p*, and *y* is realized with probability $1-p$. Participants typically choose the lottery for low certain payments. When the certain payment increases, participants switch at a given point and begin to prefer the certain payment. This is the so-called certainty equivalent, i.e., the point at which the individual is just indifferent between the certain payment and the lottery. Over 14 choice situations *x*, *y*, and *p* are manipulated. From the certainty equivalents, we estimated a standard power utility of the form $CE^\alpha = p*x^\alpha + (1-p)*y^\alpha$ for each participant, where CE is the certainty equivalent.

## Data Analysis

### Discounting Models

Exponential discounting is one model used to describe temporal behavior (Samuelson 1937). The model suggests that an outcome’s value is reduced by a constant factor for each time interval. The exponential model takes the following form:

1 $$\begin{aligned} DU(x,t)=\delta ^t U(x), \end{aligned}$$

where *DU*(*x*, *t*) is the discounted utility of outcome *x* being delivered at time *t*, $\delta$ is the discount factor (which ranges between zero and one), *t* is the period during which *x* will be delivered, and *U*(*x*) is the utility of outcome *x*. Smaller values of $\delta$ imply that the loss in value over time is larger.

Ample evidence now suggests that individual choices are time-inconsistent, and the exponential discounting model cannot account for this observation (Frederick et al. 2002). On the one hand, discount rates seem to decrease over time, such that discount rates are larger for a reward to be delivered in a year than for the same reward to be delivered in three years (Thaler 1981). On the other hand, individuals are typically more impulsive in decisions that involve the present than in decisions between two future points in time. This phenomenon has been referred to as present bias (O’Donoghue and Rabin 1999). Time inconsistencies can be described, among other approaches, by a hyperbolic or quasi-hyperbolic model. The hyperbolic model takes the following form (Mazur 1987):

2 $$\begin{aligned} DU(x,t)= \dfrac{U(x)}{1+kt}, \end{aligned}$$

where *k* is the discount rate (which is typically greater than zero) and the other notation is the same as above. Hyperbolic functions often explain temporal decisions better than exponential functions (Frederick et al. 2002). Another functional form that accounts for time inconsistencies is the quasi-hyperbolic model. The quasi-hyperbolic model takes the following form (Phelps and Pollak 1968; Laibson 1997):

3 $$\begin{aligned} \begin{aligned} \text {for } t=0,\ DU(x,t)=U(x) \\ \text {for all other } t > 0,\ DU(x,t)=\beta \delta ^tU(x) \end{aligned} \end{aligned}$$

where $\beta$ reflects the present bias, $\delta$ is the discount factor (and these two variables range between zero and one) and the other notation is the same as above. The parameters $\delta$ and $\beta$ have behavioral interpretations related to impulsivity (Altman 2015). While $\delta$ reflects the general premium that people require for delayed reward realization, $\beta$ captures the tendency whereby people are more impulsive in the short than in the long run. $\beta$ accounts for time-inconsistent behavior, i.e., for the observation that people tend to have different preferences in the long run (e.g., to eat healthy) compared to the short run (e.g., to have a chocolate bar now) (Hershfield et al. 2011; Zimbardo et al. 1997). The inability to follow one’s long-term goals results in values of $\beta$ that are smaller than one. Both parameters of the quasi-hyperbolic model have been interpreted in hot/cold terminology (Metcalfe and Mischel 1999; McClure et al. 2007) that corresponds to affective decision making in the short run ($\beta$) compared to deliberative decision making in the long run ($\delta$). Evidence exists that the two parameters have different underlying neural circuits (McClure et al. 2004).

### Model Estimations

To estimate the unknown parameters of the discounting models described above ($\delta$ , *k* and $\beta$) based on the data input from our experiment, we proceed in these steps:

- We estimate the individual risk parameter $\alpha$ for each participant based on her choices in the risk task.
- We calculate the utility of the sooner payment that was always fixed at 10 euros as $U(10) = 10^\alpha$, where $\alpha$ is the individually estimated risk parameter.
- We calculate the utility of the switching point for each of the three discounting tasks as $U(SP) = SP^\alpha$.
- Finally, we estimate the unknown parameters ($\delta$ , *k* and $\beta$) per participant such that $DU(10, t_1) \sim \ DU(SP, t_2)$, where $t_1$ is the time at which the sooner payment is delivered. In discounting tasks 1 and 2, the sooner payment is delivered in the present, i.e., $t_1$ is zero and $DU(10, t_1) = U(10)$. $t_2$ is the time at which the later payment is delivered.

The discounting models and power utility functions were estimated by using nonlinear least squares regressions with the functions *nls* of the stats package and *nlsList* of the nlme package in R (R version 3.3.2 by R Core Team 2016). The data and R-code are available at https://osf.io/sv96h/.

### Model Comparison

In the first part of the analysis, we compare the two-parameter quasi-hyperbolic discounting model to the one-parameter discounting models (exponential and hyperbolic) in terms of their ability to explain the observed data. To evaluate the performance of the different models, we compare the models’ Bayesian Information Criterion (BIC). The BIC penalizes additional free parameters more strongly than other criteria for model selection such as the Akaike Information Criterion (Kuha 2004) and therefore allows us to compare models with different degrees of freedom. Models with smaller BICs are preferred, and the strength of the evidence against the model with a higher BIC value can be summarized by the rules of thumb depicted in Table 3 (Raftery 1995).

**Table 3:** Rules of thumb for $\Delta$BIC

$\Delta$BIC	Evidence against higher BIC
0 to 2	Weak
2 to 6	Positive
6 to 10	Strong
>10	Very strong

## Power Analysis

In a meta-analysis, MacKillop et al. (2011) report an average effect size of $d=0.79$ for stronger delayed reward discounting in problem gamblers than in non-gambling controls. This effect size corresponds to an “almost large” effect as defined by Cohen (1992). A power analysis indicates that a sample of 21 (22) problem gamblers and 21 (22) controls would be needed to detect such an effect with 80% power by using a one-sided t-test (Wilcoxon-Mann-Whitney test) with alpha set to 0.05. The power analysis was performed with G*Power (Faul et al. 2007).

## Results

### Risk Preferences

In line with our previous findings (Ring et al. 2018), we find that problem gamblers, on average, are more risk-seeking than the controls, which is indicated by a larger coefficient for $\alpha$ (C group: mean = 0.93, *SD* = 0.37, median = 0.95; HG group: mean = 1.27, *SD* = 1.17, median = 0.98; PG group: mean = 2.55, *SD* = 3.63, median = 1.53). While the C group, on average, is risk-averse ($\alpha <1$), the HG and P groups are risk-seeking ($\alpha$ >1). Because the distribution of the $\alpha$ parameters in our sample violates the normality assumption (Shapiro-Wilk test, $W = 0.41, ~p < 0.001$) and we have fewer than 30 observations per group (Moffatt 2015), a (non-parametric) Kruskal-Wallis test was used to test for significant differences among the three groups. The test indicates that the groups were significantly different with respect to their $\alpha$ parameters ($H(2)=11.83, ~p =0.003$). Post hoc tests after Dunn with Bonferroni correction revealed that the PG group is significantly more risk-taking than the C group ($p = 0.002$) and the HG group ($p =0.071$). The HG and C groups did not differ significantly ($p > 0.250$).

### Model Estimations

The median estimates of the above-described discounting models are displayed in Table 4. In all three models, we find that that the PG group has a stronger preference for immediate rewards, i.e., they are more impulsive. This is indicated by a smaller value for $\delta$ in the exponential discounting model, a larger value for *k* in the hyperbolic discounting model and smaller values for $\delta$ and $\beta$ in the quasi-hyperbolic discounting model. Therefore, we find converging evidence across discounting models. Next, we will evaluate the relative quality of these models given our data and proceed with analyzing the results of the most preferred specification.

**Table 4:** Median estimates of the discounting models

	C group	HG group	PG group
$\delta$ (exponential)	0.87 [0.76; 0.91]	0.82 [0.67; 0.90]	0.57 [0.38; 0.76]
*k* (hyperbolic)	0.16 [0.10; 0.36]	0.25 [0.12; 0.56]	1.08 [0.37; 2.75]
$\beta$ (quasi-hyperbolic)	0.98 [0.98; 0.99]	0.97 [0.92; 0.99]	0.84 [0.78; 0.96]
$\delta$ (quasi-hyperbolic)	0.88 [0.79; 0.90]	0.84 [0.67; 0.90]	0.58 [0.40; 0.77]

Q1 and Q3 are in parentheses

### Model Comparison

In the overall sample, we observe very strong evidence that the quasi-hyperbolic discounting model is favored for explaining the observed data relative to the exponential and hyperbolic discounting models (see Table 5, $\Delta$BIC > 10).^2^ This suggests that introducing a second parameter that accounts for the present bias is justified due to its explanatory power. By examining how the different models perform across the different groups, we find that the previous improvement in fit is the strongest for the PG group and somewhat weaker, but still strong, for the other groups.

**Table 5:** $\Delta$BIC for different discounting models

	Overall	C group	HG group	PG group
Exponential - Quasi-hyperbolic	92.93	5.61	32.08	55.24
Hyperbolic - Quasi-hyperbolic	211.54	24.8	67.04	119.7
Hyperbolic - Exponential	102.71	19.19	34.96	48.56

### Comparison of Individual Time Preferences Across Groups

**Fig. 1:**
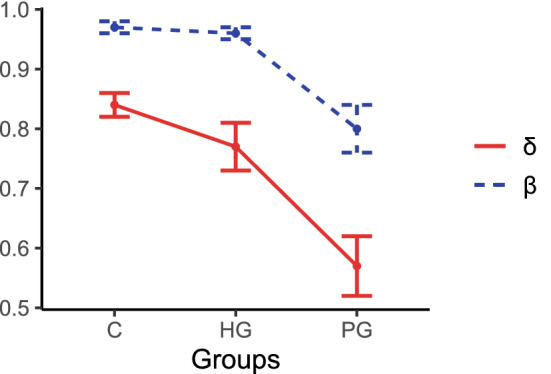
Mean $\delta$ and $\beta$ by group. The error bars indicate the standard errors of the mean

Having shown that the quasi-hyperbolic discounting model is preferred for explaining the data, we compare the temporal preferences across groups within this parametrization. Our results for $\delta$ and $\beta$ for the different groups are displayed in Figure 1. Because the distributions of $\delta$ and $\beta$ violate the normality assumption (Shapiro-Wilk tests, $p<0.001$), and each group contains fewer than 30 observations (Moffatt 2015), non-parametric tests (Kruskal-Wallis tests and subsequent post hoc tests after Dunn with Bonferroni correction) were performed to identify significant differences among the groups. The Kruskal-Wallis test indicates that the groups were significantly different with respect to their long-term discount factor $\delta$ ($H(2)=16.61, ~p<0.001$). The post hoc tests after Dunn with Bonferroni correction revealed a significant difference between the PG and C groups and between the PG and HG groups (PG versus C group: $p<0.001$; PG versus HG group: $p=0.016$; C versus HG group: $p >0.250$). For the parameter $\beta$, a Kruskall-Wallis test indicates that the groups were significantly different ($H(2)=22.75, ~p <0.001$). Post hoc tests after Dunn with Bonferroni correction revealed a significant difference between the PG and the C groups and between the PG and the HG groups (PG versus C group: $p<0.001$; PG versus HG group: $p=0.003$; C versus HG group: $p >0.250$).^3^

**Fig. 2:**
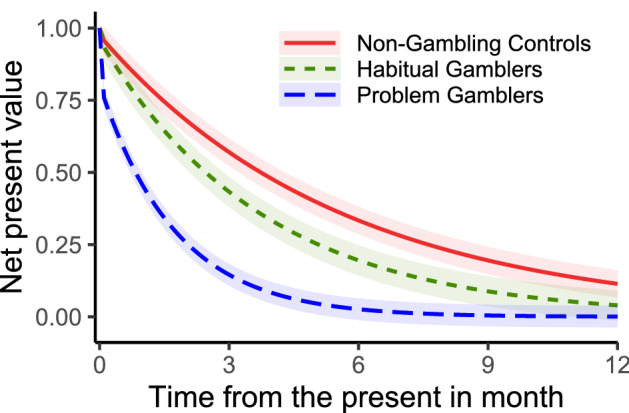
Mean net present value over time by group. The shaded areas indicate the standard errors of the mean

We also run ordinary least squares regressions to study the effect of the SOGS scores on $\delta$ and $\beta$. Table 6 reveals that both $\delta$ and $\beta$ show a significant negative correlation with the participants’ SOGS scores ($p<0.001$ for both parameters). For illustrative purposes, Figure 2 plots the quasi-hyperbolic discounting model based on the mean values of $\delta$ and $\beta$ for each group. The significantly smaller $\beta$ in the PG group leads to a deeper initial jump, while the significantly smaller $\delta$ additionally leads to a faster loss in value over time that is independent of the initial jump.

**Table 6:** Regression analysis of $\beta$ and $\delta$

Dep. var.	$\beta$	$\delta$
(Intercept)	$$1.019^{***}$$	$$0.878^{***}$$
	(0.065)	(0.096)
SOGS	$$-0.017^{***}$$	$$-0.027^{***}$$
	(0.004)	(0.005)
Male	$-0.019$	$-0.021$
	(0.043)	(0.063)
Age	$-0.0005$	$-0.001$
	(0.001)	(0.002)
R$^2$	0.248	0.262
Num. obs.	74	74

$$^{***}p<0.001$$

In the overall sample, there is a strong and statistically significant positive correlation between the individual $\delta$ and $\beta$ parameters ($r(72)=0.71, ~p<0.001$). By examining the different groups, we find that the positive correlation is statistically significant for the PG group ($r(23)=0.79, ~p<0.001$), while it is not statistically significant for the HG group ($r(21)=0.07, ~p >0.250$) and the C group ($r(24)=0.19, ~p >0.250$).

## Discussion

In this paper, we study temporal discounting in problem gambling. While previous research has commonly reported that problem gamblers have a stronger preference for smaller immediate rewards than do non-gambling controls, we apply the quasi-hyperbolic discounting model to disentangle two different aspects of impulsivity. These aspects are a short-term oriented aspect of impulsivity, which is typically called the present bias, and a long-term oriented aspect of impulsivity, the long-run discount factor. In the first part of the analysis, we observe that the quasi-hyperbolic model is strongly preferred for explaining the data, mainly due to its explanatory power for the PG group. Within this parametrization, we find that problem gamblers have a stronger present bias and a smaller long-term discount factor than the controls. Furthermore, both parameters correlate significantly with the participants’ SOGS scores as a continuous measure of gambling behavior. Both parameters add to the general finding that problem gamblers discount delayed rewards more rapidly than do non-gambling controls. While differences in risk attitudes between problem gamblers and non-gambling controls are expected due to the nature of gambling activities (Ligneul et al. 2012), the differences in temporal choices—at first glance—appear less intuitive. Different theories, however, have been developed that link temporal choices to gambling behavior.

Gambling has severe negative long-term effects, such as social isolation (Trevorrow and Moore 1998) and financial problems (Pietrzak et al. 2005). One theory that links time to risk preferences suggests that problem gamblers might discount these long-term effects more heavily than do non-gambling controls. In this case, the disutility of the negative long-term consequences is smaller and might fail to deter problem gamblers from gambling (Petry and Madden 2010). Similar mechanisms seem to be at work in drug-addicted individuals (Odum et al. 2000, 2002). Within the quasi-hyperbolic model, this theory would be reflected by a long-term discount factor that is smaller for problem gamblers than for non-gambling controls. We find evidence for this theory in our data, because we observe that problem gamblers have a smaller long-term discount factor than do non-gambling controls. This might indicate that the former discount the negative long-term consequences more heavily. Note, however, that we analyze the gain domain only, and evidence suggests that discounting patterns differ between gains and losses (Appelt et al. 2011).

Moreover, the present bias might be more pronounced in problem gamblers (Petry and Madden 2010). According to this theory, gambling is pursued because individuals overvalue the immediate satisfaction from this activity, e.g., the thrill of gambling, relative to the above-outlined negative long-term consequences. Within the quasi-hyperbolic model, this would be reflected by a stronger present bias, i.e., a smaller $\beta$, without systematic differences in the long-run discount factor. We also find evidence for this theory, because we observe that problem gamblers have a significantly greater present bias than do non-gambling controls. Note that different types of gambling have different payment dates. While payments are made directly in casinos, this is not the case for online gambling, where bank transfers might take a few days, or weekly lotteries. Non-direct payments should therefore be less attractive for individuals with a strong present bias. Since we do not differentiate between different types of gambling, and our sample is too small for such an analysis establishing this link is left for future research.

## Footnotes

1 Further details about the risk task can be found in Vieider et al. (2015), and further details about the data in Ring et al. (2018).

2 Please note that for one participant, the exponential discounting model did not converge.

3 All results remain stable if we use parametric tests (ANOVA and subsequent t-tests with Bonferroni correction).
